# Targeting glutamine uptake in AML

**DOI:** 10.18632/oncoscience.1

**Published:** 2014-01-10

**Authors:** Nathalie Jacque, Didier Bouscary

**Affiliations:** ^1^ Unité Fonctionnelle d'Hématologie, Hôpital Cochin, AP-HP. Paris, France.; ^2^ Institut Cochin, Département d'Immuno-Hématologie, CNRS UMR8104, INSERM U1016. Paris, France.; ^3^ Université Paris Descartes, Faculté de Médecine Sorbonne Paris Cité.

Cancer cells require nutrients and energy to adapt to increased biosynthetic activity and depend on mitochondrial oxidative phosphorylation (OXPHOS) and glycolysis. Whereas they exhibit a pronounced Warburg effect, their TCA cycle remains intact and becomes more dependent on glutamine metabolism through glutaminolysis[1]. Besides this role, intracellular glutamine is also essential for mTORC1 activation by leucine[2]. Many upstream signals regulate mTORC1 activation. Among them, a major process is the availability of leucine, which is required to activate the Rag (for Ras-related GTPases) proteins that enable the proper localization of mTORC1 at the lysosome surface close to its activator Rheb[3]. Leucine uptake into the cells is regulated by the bidirectional transporter SLC7A5/3A2, in exchange for glutamine. The level of leucine thereby depends on the intracellular glutamine concentrations, which is mainly mediated by the high affinity transporter SLC1A5. Thus, the cellular uptake and subsequent rapid efflux of glutamine in the presence of leucine make glutamine availability a limiting step for the activation of mTORC1. MTORC1 positively regulates protein translation through phosphorylation of protein S6 Kinase (P70S6K) and eukaryotic initiation factor 4E (eIF4E)-binding protein 1 (4E-BP1). Protein synthesis is controlled by the translational repressor 4E-BP1 whose phosphorylation at serine 65 is required to initiate the formation of the translation initiation complex.

**Figure:**
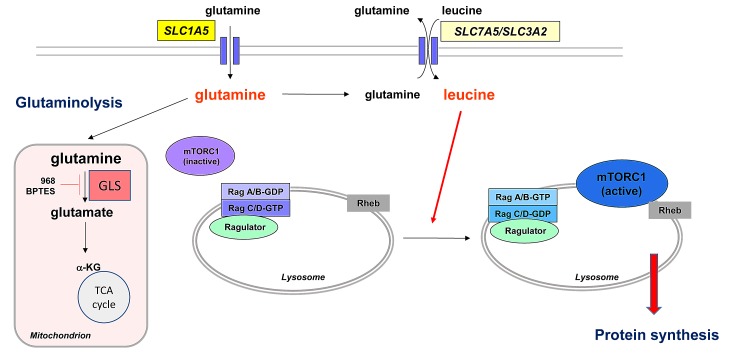
AML cells are addicted to glutamine Limiting glutamine uptake by L-asparaginase, glutamine removal or knockdown of the glutamine transporter SLC1A5 inhibits mTORC1 activity and protein synthesis by limiting leucine uptake by the bidirectional transporter SLC7A5/3A2. Resulting intracellular leucine depletion limits the proper localization of mTORC1 close to its direct activator, the Rheb kinase, at the lysosomal surface. Limiting glutamine uptake may also exert anti-leukemic effects through inhibition of glutaminolysis, the key gatekeeper of which is the enzyme glutaminase (GLS) which catalyzes the hydrolysis of glutamine to glutamate. Some GLS isoforms can be specifically inhibited by compound 968 and BPTES.

The dependence of acute myeloid leukemia (AML) cells to glutamine is little studied. In a recent work, we have tested the effects of glutamine depletion in AML cells[4]: leukemic cells are sensitive to glutamine removal leading to mTORC1 inhibition and apoptosis. The drug L-asparaginase (L-ase) also inhibits mTORC1 activity in AML cells, suppresses protein synthesis and induces apoptosis. The anti-leukemic effects of the two clinically available forms of L-ase, *E Coli* L-ase (Kidrolase®) and *E. Chrysanthemi* L-ase (Erwiniase®) are not mediated by the asparaginase activity of the enzyme. L-ases have also a glutaminase activity and transform extracellular glutamine into glutamate. Both L-ases induce dose and time-dependent mTORC1-inhibition which correlates with extra-cellular glutamine depletion[4]. Downstream of mTORC1, L-ase suppresses 4E-BP1 phosphorylation and inhibits [S^35^] methionine incorporation into newly synthesized proteins, indicative of global protein synthesis inhibition. Finally, we showed that L-ase treatment acts through inhibition of leucine entry into the cells by depleting intracellular glutamine, thus preventing mTORC1 activation by Rheb at the lysosomal surface[4].

Overall, those data open the way for new therapies targeting glutamine uptake in AML. L-ase has been previously tested in AML. A randomized study that compared the effects of high dose cytarabine with or without *E. Coli* L-ase in refractory/relapsed AML in adults revealed a significantly increased CR rate with the association (40% vs. 24%)[5]. Blast cells from the M1, M4 and M5 FAB subtypes may display an *in vitro* sensitivity to L-ase very close to that of acute lymphoblastic leukemia cells. Further preclinical studies are thus warranted to characterize new chemotherapeutic combinations in association to L-ase in AML.

We also identified potential mechanisms of resistance to L-ase treatment in AML. L-ase upregulates glutamine synthase (GS) protein expression and this process may limit the L-ase-induced anti-leukemic activity in AML. GS inhibition by the L-methionine-sulfoximine (MSO) may increase the sensitivity of AML cells to L-ase as described in ALL. Moreover, L-ase triggers an autophagic process, probably resulting from mTORC1 inhibition (mTORC1 inhibits autophagy by controlling the activity of Atg1/ULK and ATG13 proteins). Knockdown of either ATG5 or Beclin, two proteins controlling autophagy, increase apoptosis in L-ase-treated AML cells. The future testing of a combination of autophagy inhibitors and L-ase is thus warranted in AML.

Another therapeutic strategy to exploit AML glutamine dependence may be to target the glutamine transporters. We showed that the knockdown of the high affinity transporter SLC1A5 has strong anti-leukemic effects in vitro and inhibits tumor formation in an AML mouse xenotransplantation model[4]. However, SLC1A5 inhibition was not efficient in all AML cell lines, indicating that other glutamine transporters may control glutamine-mediated mTORC1 activation in AML[4].

We only address here the role of glutamine uptake by AML cells on the control of mTORC1 activation. Studies are needed to examine its role in mitochondrial anaplerosis through glutaminolysis. The key gatekeeper of glutaminolysis is the enzyme glutaminase (GLS) which catalyzes the hydrolysis of glutamine to glutamate, which is then converted to the TCA cycle intermediate α-ketoglutarate (α-KG). Two different forms of GLS exist: the liver-type glutaminase (LGA, coded by the GLS2 gene) and the glutaminase 1 (GLS gene) which exists as two splice variants: the longer form called kidney-type glutaminase (KGA) and the shorter called glutaminase C (GAC)[6]. The two GLS1 isoforms are up-regulated in cancers. Two inhibitors of GLS1 have been characterized: the compound 968, a specific allosteric inhibitor of GAC, and BPTES that potently inhibits both GLS isoforms. Although several studies showed anti-tumoral effects of the two inhibitors in many cancers[7], the targeting of glutaminolysis in AML is still to be proved. Recently, it has been suggested that BPTES could suppress the growth of primary AML cells with IDH mutations[8].

To conclude, through inhibition of mTORC1 activity and/or through suppression of the mitochondrial TCA cycle, glutamine metabolism constitutes an appealing target for treating this particularly poor prognosis disease.
